# Validity of the Demirjian Method for Dental Age Estimation in Romanian Children

**DOI:** 10.3390/children9040567

**Published:** 2022-04-16

**Authors:** Abel Emanuel Moca, Gabriela Ciavoi, Bianca Ioana Todor, Bianca Maria Negruțiu, Emilia Albinița Cuc, Raluca Dima, Rahela Tabita Moca, Luminița Ligia Vaida

**Affiliations:** 1 Department of Dentistry, Faculty of Medicine and Pharmacy, University of Oradea, 10 Piața 1 Decembrie Street, 410073 Oradea, Romania; abelmoca@yahoo.com (A.E.M.); biancastanis@yahoo.com (B.M.N.); cucalbinita@yahoo.com (E.A.C.); razdima@gmail.com (R.D.); ligia_vaida@yahoo.com (L.L.V.); 2 Doctoral School of Biomedical Sciences, University of Oradea, 1 Universității Street, 410087 Oradea, Romania; rahelamoca@gmail.com

**Keywords:** dental age, Demirjian method, Romanian children

## Abstract

Dental age assessment is useful in various medical fields. The Demirjian method for dental age estimation is one of the most widely used in the field of pediatric dentistry. The aim of this study was to verify the accuracy of the Demirjian method in determining age in a sample of girls and boys from Oradea, Romania. This retrospective and radiographic study was based on the evaluation of 1006 panoramic radiographs, belonging to 1006 patients (431 boys and 575 girls) with ages between 3 and 13.9 years from the city of Oradea, Romania. They were collected from three private dental practices from Oradea and were analyzed between 1 September 2021, and 10 November 2021. The patients were distributed into 11 age groups, each group spanning over one year (e.g., 3–3.9, 4–4.9). For the assessment of dental age, the Demirjian method was used, which is based on the evaluation of the developmental stages of the lower left permanent teeth. The mean chronological age of the patients was 9.496 ± 2.218 years, and the mean dental age was 10.934 ± 2.585 years. The overestimation of dental age was obtained in all age groups for the entire sample. As such, dental age was higher than chronological age, with values varying from 0.34 years in the 3–3.9 years age group to 1.7 years in the 10–10.9 years age group. In girls, dental age was higher than chronological age, with values varying from 0.46 years in the 3–3.9 years age group to 1.73 years in the 11–11.9 years age group, while in boys, the values varied from 0.15 years in the first age group to 2.02 years in the 10–10.9 years age group. The comparison of the differences between chronological age and dental age according to the gender of the patients revealed that the distribution of the differences was nonparametric in both groups according to the Shapiro–Wilk test (*p* < 0.05). The differences between the groups were not significant according to the Mann–Whitney U test. However, larger differences were identified for boys (1.46 years) than girls (1.417 years), with an overestimation of the dental age. The Demirjian method overestimated the age of the children included in the investigated sample, with different values for the different age groups investigated, and requires adaptations.

## 1. Introduction

Knowing the accurate age of a child is essential for an optimal choice of a specific therapeutic approach, and it is useful in various medical fields, such as orthodontics, pediatric dentistry, and endocrinology, mainly due to its ability to influence the ideal moment for beginning different medical treatments [1]. In forensic medicine, age estimation is mainly needed in cases where the patient’s identity is unknown and the patient’s real age cannot be proven with valid documentation [2]. In order to rightly assess the development of a patient, various techniques for determining age have been imagined over time. They are based on the skeletal development of the hand and wrist [3], on the skeletal development of the cervical vertebrae [4], on dental maturation [5], and on the development of secondary sexual characteristics [6].

In pediatric dentistry, the most accessible methods for age estimation are methods that assess the skeletal development of the patient based on the morphology of the cervical vertebrae [4], as well as methods that are based on the assessment of dental development [5]. The appearance of the cervical vertebrae can be visualized with the help of lateral cephalometric radiographs, which are necessary for establishing a correct orthodontic diagnosis [4]. The best-known technique for staging cervical vertebral development is the cervical vertebral maturation (CVM) method proposed by Baccetti et al. (2005) [7]. Methods based on assessing dental development can be effortlessly used [8], most of them requiring a simple radiographic examination, which allows a proper visualization of all existing teeth, regardless of their intraoral or intramaxillary presence [9]. Over time, several methods have been used to estimate dental age. The Nolla method is based on the existence of 10 stages of dental development, which can be established on panoramic radiographs [10]. The Cameriere method is based on measuring the open apices of young permanent teeth on panoramic radiographs [11], and the Willems method is an adaptation of the Demirjian method [12].

The Demirjian method is one of the most used for estimating a child’s dental age [13], mainly due to its simplicity. It was developed on a French-Canadian sample population, and it is based on the analysis of the erupted or unerupted permanent teeth located on the lower left dental arch [14]. It has been widely applied and adapted to various populations [15,16]. Dental age can be correlated with the skeletal age, in order to monitor the symmetry between skeletal and dental development [17], as well as with the chronological age, in order to validate or invalidate the method in the studied population [1].

Assessing a child’s dental age is important for determining the course of treatment and the development of the dentition. Although the Demirjian method for age determination was applied and tested in many populations, the obtained results may vary, and it needs to be validated in the desired population sample. In the Romanian population, the need for an appropriate method for dental age assessment was considered necessary.

The aim of this study was to verify the accuracy of the Demirjian method in determining age in a sample of girls and boys from Oradea, Romania. The goal was to establish the dental age of the patients using the Demirjian method. After estimating the dental age, it was compared with the chronological age, in order to verify if the two correspond or if the Demirjian method underestimates or overestimates the age of the patients from the studied sample.

## 2. Materials and Methods

### 2.1. Ethical Considerations

The research was carried out in conformity with the 1964 Declaration of Helsinki and its subsequent amendments and was approved by the University of Oradea’s Research Ethics Committee (No. CEFMF/02 from 21 October 2021).

### 2.2. Sample Selection

This investigation was conducted as a retrospective and radiographic study using digital panoramic radiographs from a group of patients from the city of Oradea, North-Western Romania. The analysis of panoramic radiographs was performed between 1 September 2021, and 10 November 2021. Three private dental practices in Oradea, Romania, provided the panoramic radiographs. They were deemed necessary for undertaking pedodontic or orthodontic treatments and were not requested solely for this study. The Soredex Cranex Novus Panorex (Soredex, Milwaukee, WI, USA) equipment was used for all of the radiographs.

After the initial examination, a recommendation for a panoramic radiograph was made for each patient that required further treatment, and all caregivers and parents signed informed consent and agreed that the radiographs belonging to underaged patients could be used for future studies. All radiographs were dated and contained the patient’s name and date of birth. All patients that were initially selected underwent a panoramic radiological examination between 1 January 2018, and 1 June 2021. The selected panoramic radiographs were digital and saved as a Joint Photographic Experts Group (JPEG) image.

The following inclusion criteria were further applied: panoramic radiographs belonging to individuals with ages between 3 and 13.9 years; patients who required a panoramic radiograph for the diagnosis and treatment of dental diseases; patients who had a known date of birth; and patients who had a known date of the radiological assessment.

An initial number of 1145 panoramic radiographs, corresponding to the aforementioned inclusion criteria was selected.

The following exclusion criteria were applied: panoramic radiographs belonging to uncooperative patients whose behavior prevented the radiological examination from being completed, resulting in an indistinct radiographic image (*n* = 23); patients who benefited from an orthodontic treatment before taking the panoramic radiography (*n* = 16); patients who were undergoing an orthodontic treatment when the radiographic image was taken (*n* = 13); patients from another country (*n* = 9); patients with local or systemic diseases that could influence dental development (*n* = 27). All radiographs that were unclear and did not permit the proper investigation of the lower left permanent teeth were excluded as well (*n* = 51). After applying the exclusion criteria, 139 panoramic radiographs were eliminated.

A final number of 1006 radiographs remained for the completion of this study. The remaining patients were distributed into 11 age groups. Each age group covered one year (e.g., 3–3.9 years; 4–4.9 years). The one year per age group span was selected in order to have a more accurate representation of the mean values for chronological age and dental age and for the differences between the two.

### 2.3. Sample Size Calculation

A sample size estimation was made using GPower 3.1.9.7 software (Heinrich Heine University, Düsseldorf, Germany). By the design of the study, it was considered that the measured score will be compared between age groups (11 groups in this study) using one-way ANOVA tests or Kruskal–Wallis H tests. We calculated an effect size equal to f = 0.4277. Therefore, it was estimated using an effect size of f = 0.4277, with a minimum power of 0.8 and an α = 0.05, that the minimum total sample size should be 99 radiographs for an ANOVA test between 11 groups. However, we wanted to include in this study all the available panoramic radiographs that matched the inclusion criteria. This is why the initial number of radiographs included in this research was 1145, and the final number of radiographs was 1006. This number was over the minimum amount required for a statistical power of 0.8.

### 2.4. Chronological Age and Dental Age Assessment

By subtracting the patient’s date of birth from the date of the radiograph, the chronological age was determined.

**Figure 1:**
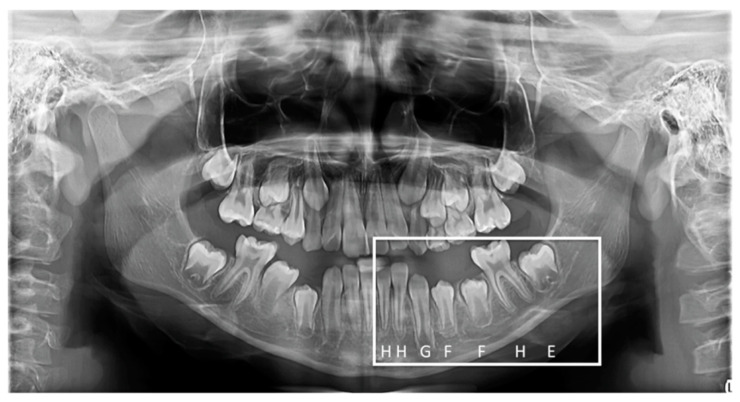
Dental age assessment using the Demirjian method.

The Demirjian method for estimating dental age was used to assess patients’ age. This method involves the analysis of panoramic radiographs of the 7 lower left permanent teeth (Figure 1). The third permanent molar is not taken into consideration. This method takes into consideration 8 stages of tooth development, noted with letters from A (the lowest maturation point) to H (the highest maturation point). These stages (noted as letters) will be allocated a numerical value based on the tables provided by Demirjian et al. (1974) [14], values that are different for boys and girls. The values are summed once the 7 values corresponding to the 7 lower left permanent teeth are achieved, and the final result is represented by the Dental Maturation Score, which will be turned into dental age using the tables envisioned by the method’s creators [14].

A single investigator (A.E.M.) examined the panoramic radiographs for the purpose of determining dental age, in order to avoid any interoperator bias. After 30 days from the initial analysis of the panoramic radiographs, to measure intrarate agreement in this database, for every tenth patient, the assessment of dental age was repeated by the same investigator, and in the analysis were included 100 patients with the dental age examined twice. The intraclass coefficient (ICC) value on the average measures of the dental age was 0.996 (95% C.I.: 0.995–0.998), representing an excellent intrarate agreement for the assessment of the dental age.

### 2.5. Statistical Analysis

IBM SPSS software, version 20 (IBM, Chicago, IL, USA) was used for statistical analysis. The Shapiro–Wilk test was used to determine the distribution of quantitative data, which were expressed as mean values with standard deviations (or medians with interpercentile intervals, depending on the distribution), while categorical variables were expressed in absolute or percentage form. The Mann–Whitney U or Kruskal–Wallis H test was used to evaluate the independent quantitative variables because their distribution was nonparametric. The Kruskal–Wallis H test was used in order to evaluate the differences between chronological age and dental age for the various age groups for the entire sample or for selected genders. The Mann–Whitney U test was used in order to evaluate the differences between chronological age and dental age in boys and girls.

For the analysis of the intrarate agreement an intraclass correlation coefficient (based on a two-way mixed with absolute agreement model) was made to measure the reliability between the two assessments.

## 3. Results

In this study, a total of 1006 panoramic radiographs were analyzed, of which 431 radiographs belonged to boys and 575 radiographs to girls. Data in Table 1 show the distribution of the patients according to the different age groups. Most of the patients were distributed in the 8–8.9 years (18.9%), 9–9.9 years (15.7%), and 7–7.9 years (14%) age groups. In the girls sample, it was observed that the largest number of patients was distributed in the 8–8.9 years age group (99 patients), as observed for the boys sample as well, with 91 patients being distributed in the same age category. The fewest patients for boys and girls were distributed in the first age group (3–3.9 years).

The mean chronological age of the patients was 9.496 ± 2.218 years, with a median of 9.3 years and a range between 3 and 13.9 years. The mean dental age, estimated by using the Demirjian method, was 10.934 ± 2.585 years, with a median of 10.8 years and a range between 3 and 16 years.

Data in Table 2 show the comparison of the differences between chronological age and dental age according to the different age groups. The differences were distributed in a nonparametric manner in most age groups according to the Shapiro–Wilk test (*p* < 0.05). The Kruskal–Wallis H test showed that the differences between the analyzed groups were significant (*p* = 0.010).

**Figure 2:**
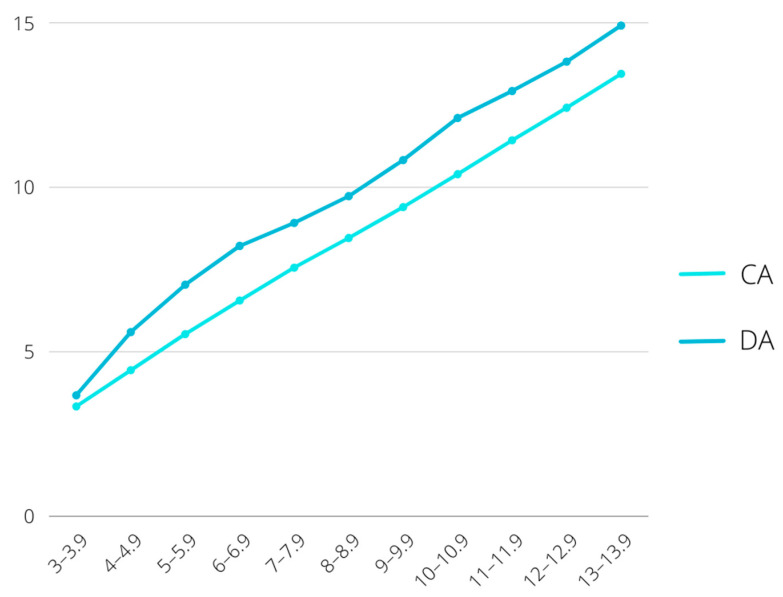
Chronological age (CA) and dental age (DA) in different age groups.

Figure 2 emphasizes the differences between the values obtained for the chronological age and dental age.

The mean chronological age of girls was 9.65 ± 2.211 years, and the mean chronological age of boys was 9.28 ± 2.213 years. The mean dental age of girls was 11.07 ± 2.6 years, while the mean dental age of boys was 10.74 ± 2.55 years. The comparison of the differences between chronological age and dental age according to the gender of the patients revealed that the distribution of the differences was nonparametric in both groups according to the Shapiro–Wilk test (*p* < 0.05). The differences between the groups were not significant according to the Mann–Whitney U test. However, larger differences were identified for boys (Table 3).

Data in Table 4 show the comparison of the differences between the chronological age and dental age according to the different age groups for boys and girls. The differences had a nonparametric distribution in most age groups in both girls and boys according to the Shapiro–Wilk test (*p* < 0.05). The Kruskal–Wallis H test showed that the differences between the analyzed groups were significant in girls (*p* < 0.001) but were not significant in boys (*p* = 0.152).

## 4. Discussion

Dental age estimation remains important because it is probably the safest and simplest method for assessing the age of a child or of a young adult [18]. Dental age assessment is not only useful in pediatric dentistry and orthodontics but can be used by immigration services for international adoptions when a birth certificate is not available or when the date of birth is not known and cannot be determined [18]. It can also be used for identifying victims of natural disasters and for other circumstances when an individual’s date of birth is unknown [19]. Identifying a method that rightly assesses age is therefore important for every population globally.

Dental age can be determined by morphological, biochemical, and radiological methods [20]. Morphological methods for dental age estimation are generally derived from the method originally conceived by Gustafson [21] and involve the ex vivo microscopic analysis of extracted teeth [20]. Morphological methods raise major ethical issues when it comes to their application to living individuals. At best, they could be used postmortem, but even then, ethical, religious, or cultural issues may arise [20]. Biochemical methods for dental age assessment are based on the analysis of the level of D-aspartic acid in enamel, dentin, and cement, which increases with age [20]. The biochemical method proposed by Ritz et al. (1995) allows a biopsy to be performed on the dentin, thus excluding the need for tooth extraction in order to identify the age of a living person [22]. Finally, radiographic methods are numerous and noninvasive to the dental structure. Through these methods, dental age can be estimated in prenatal, neonatal, postnatal, in children and adolescents, and in the adult population [20]. Although there are multiple methods of determining dental age based on the radiological examination, such as the Nolla [10], Cameriere [11], or Willems [12] method, one of the most widely used and popular methods is the Demirjian method [14].

In this study, dental age was estimated by the Demirjian method due to the fact that it is one of the best known and used methods for estimating dental age [13]. One of the advantages of this method is that it allows the use of panoramic radiographs to estimate dental age and does not require other invasive methods.

Panoramic radiographs are widely used in dentistry and pediatric dentistry and are important in establishing a correct diagnosis [17]. In fact, panoramic radiography is the most recommended screening method in dentistry [23] because it allows the visualization of the maxillary and mandibular region on a single film, including the erupted or nonerupted teeth [24]. A final number of 1006 panoramic radiographs were used in this study to estimate dental age.

The Demirjian method was developed on a French-Canadian population sample, but it has been vastly applied and verified in other populations around the globe, correctly estimating, underestimating, or overestimating dental age.

The overestimation of dental age by the Demirjian method was obtained in some European populations. Urzel and Bruzek (2013) compared four methods of estimating dental age in a group of French children, including the Demirjian method, which overestimated the dental age with an average of 0.45 years for girls and 0.46 years for boys [25]. In our study, only the Demirjian method was used for dental age estimation, but the overestimation was higher, with an average of 1.4 years for girls and 1.5 years for boys. In Germany, the assessment of age by the Demirjian method overestimated dental age, with an average of 0.46 years for boys and 0.55 years for girls, results similar to the study applied to French children [26]. The sample size used for dental age assessment in the German population was 951 panoramic radiographs (in the first sample) [26], and this was similar to this study, where the sample consisted of 1006 panoramic radiographs. A study performed on a Spanish population, which used two different methods for dental age estimation, Nolla and Demirjian, reported an overestimation of dental age by the Demirjian method, with an average of 0.987 years for boys and 0.718 years for girls [27]. However, the Spanish population study was performed on a total of 2641 panoramic radiographs [27], which is more than double the study performed on the Romanian population. Another difference is the age of the participants. In the Spanish study, the age range was between 7 and 21 years, and the participants were distributed into three age groups: under 14 years, from 14 to 18 years, and over 18 years [27]. In our study, the age range was between 3 and 13.9 years, and the patients were distributed into 11 age groups. The inferior limit of 3 years was selected because it is the lowest age that can be assessed by the use of the Demirjian method [14]. The superior limit of 13.9 years was selected because apices of second permanent molars, usually the last to erupt on the left dental arch, are expected to close at around 14 years and 9 months for girls and 15 years and 5 months for boys [28]. Due to the distribution of the patients in various age groups, the 13.9 years age limit was established.

Different values of overestimation of the dental age have also been found in children populations from Macedonia [29] and Turkey [30]. A study performed in Macedonia on 966 panoramic radiographs reported an overestimation of 1.12 years for girls and 1.07 years for boys by the Demirjian method for dental age estimation [29]. These results are very similar to those obtained in our study sample, and the sample size is similar as well. In a Turkish population, a study was conducted on 635 panoramic radiographs, and the overestimations obtained varied from 0.10 to 0.76 years for boys and 0.28 to 0.87 years for girls [30]. In the present study, the overestimations varied from 0.46 to 1.73 years for girls and from 0.15 to 2.02 years for boys, values that are higher than those obtained in the Turkish population.

In contrast with this study and with other studies conducted on various European populations in which the Demirjian method overestimated the dental age, there are studies in which this method underestimated the dental age. Sobiseka et al. (2018) applied the Demirjian method and compared it to the Willems method to estimate dental age on a sample of 1002 panoramic radiographs. The size of the sample, as well as the distribution of patients according to gender, is similar to the present study conducted on the Romanian population. The study was conducted on a Polish population and revealed underestimations of the patients’ age, with an average of 0.317 years [31]. These results are different from the results obtained in the present study, where an overestimation of the dental age was obtained in all age groups for boys and girls.

Given the fact that the Demirjian method tends to either overestimate or underestimate the dental age, some authors have made adaptations of the original Demirjian values in their studied populations. Chaillet et al. (2004) obtained a high accuracy in the estimation of dental age in a Belgian population after adapting the Demirjian scores and using Belgian weighted scores [32]. In Switzerland, Birchler et al. (2016) applied the Demirjian method but made some changes to the original method. Compared to the original method, the authors staged not only the left lower permanent teeth but also the upper left permanent teeth, as well as the third permanent molars, obtaining a good estimation of the patients’ age [33]. Given the results of this study, it can be considered that the Romanian population from this study sample requires an adaptation of the Demirjian method. The development of a new model based on the values obtained in this study could be a starting point. Although this study was conducted on a large sample (1006 panoramic radiographs) and obtained overestimations of the dental age by using the Demirjian method, it has its limitations.

The limitations of this study are, firstly, given by the application to a somewhat geographically restricted sample. The study was applied in Oradea, North-Western Romania, and did not include other areas in Romania. Applying the Demirjian method in other Romanian regions and setting up a unitary sample would be beneficial for future research. The radiographs included in the study were collected from three private dental practices and were considered necessary for undergoing a pedodontic or orthodontic treatment. This aspect determined the exclusion of patients who presented excellent oral health. However, the burden of dental caries in Romania is a real issue. Tudoroniu et al. (2020) recently reported a caries prevalence of 95.5% in the Romanian adolescent population [34]. Another study reported the caries experience among Romanian schoolchildren as being over 80% [35]. This is one of the reasons for which assessing dental age on caries-free Romanian children population was not considered necessary since most of the children are affected by dental caries and will require dental treatment. Another reason for which the sample was limited to future pedodontic or orthodontic patients was the need for panoramic radiographs for dental age assessment. Exposing children that are not in need of dental treatment to useless radiation was considered unethical. Another limitation that has to be considered is that in the first three age groups (3–3.9 years, 4–4.9 years, 5–5.9 years) the subject number was too small for a proper statistical inference. Additionally, for a better workflow, computerized methods for assessing dental age could be used instead of the classic assessment, which involves the direct examination of the panoramic radiographs.

We consider that this study opens the possibility of continuing the investigation of dental age in Romania using the Demirjian method but also other dental age estimation methods, as well as the adaptation of the Demirjian method in the Romanian population.

## 5. Conclusions

The Demirjian method overestimated the age of children from Oradea, Romania, who were included in this study, with different values of overestimation for the different age groups investigated. The highest value of the differences was obtained for the 13–13.9 years age group and the lowest value for the first age group (3–3.9 years). The overestimation of the dental age interested both girls and boys. An adaptation of the Demirjian method for the Romanian population or assessing dental age by other dental age assessment methods would be beneficial.

## Figures and Tables

**Table 1:** Distribution of the patients in different chronological age groups.

Age Group (in Years)	Girls (*n*, %)	Boys (*n*, %)	Total (*n*, %)
**3–3.9**	3 (0.30%)	2 (0.20%)	5 (0.5%)
**4–4.9**	9 (0.89%)	10 (0.99%)	19 (1.9%)
**5–5.9**	13 (1.29%)	12 (1.19%)	25 (2.5%)
**6–6.9**	34 (3.38%)	32 (3.18%)	66 (6.6%)
**7–7.9**	74 (7.36%)	67 (6.66%)	141 (14%)
**8–8.9**	99 (9.84%)	91 (9.05%)	190 (18.9%)
**9–9.9**	92 (9.15%)	66 (6.56%)	158 (15.7%)
**10–10.9**	75 (7.46%)	34 (3.38%)	109 (10.8%)
**11–11.9**	68 (6.76%)	53 (5.27%)	121 (12%)
**12–12.9**	66 (6.56%)	39 (3.88%)	105 (10.4%)
**13–13.9**	42 (4.17%)	25 (2.49%)	67 (6.7%)

*n*, number; %, percentage.

**Table 2:** Chronological age, dental age, and differences between the two.

Age Group (in Years)	CA with SD (in Years)	DA with SD (in Years)	CA-DA with SD (in Years)	*p* *
3–3.9 (*p* = 0.823 **)	3.34 ± 0.29	3.68 ± 0.43	−0.34 ± 0.57	0.010
4–4.9 (*p* = 0.013 **)	4.44 ± 0.29	5.6 ± 1.32	−1.15 ± 1.19	
5–5.9 (*p* = 0.505 **)	5.54 ± 0.27	7.04 ± 0.46	−1.49 ± 0.4	
6–6.9 (*p* < 0.001 **)	6.56 ± 0.29	8.22 ± 0.79	−1.66 ± 0.76	
7–7.9 (*p* < 0.001 **)	7.56 ± 0.28	8.92 ± 1.06	−1.36 ± 1	
8–8.9 (*p* < 0.001 **)	8.46 ± 0.30	9.73 ± 1.21	−1.27 ± 1.18	
9–9.9 (*p* = 0.777 **)	9.40 ± 0.30	10.83 ± 1.24	−1.42 ± 1.18	
10–10.9 (*p* < 0.001 **)	10.40 ± 0.29	12.11 ± 1.22	−1.7 ± 1.16	
11–11.9 (*p* = 0.070 **)	11.43 ± 0.29	12.93 ± 1.46	−1.5 ± 1.46	
12–12.9 (*p* = 0.006 **)	12.42 ± 0.29	13.82 ± 1.64	−1.4 ± 1.59	
13–13.9 (*p* < 0.001 **)	13.45 ± 0.32	14.92 ± 1.56	−1.46 ± 1.5	

CA, chronological age; DA, dental age; SD, standard deviation; * Kruskal–Wallis H Test, ** Shapiro–Wilk Test.

**Table 3:** Comparison of the differences between chronological age and dental age according to the gender of the patients.

Gender	Mean Age ± SD	Median (IQR)	*p* *
Girls (*p* < 0.001 **)	−1.417 ± 1.2	−1.4 (−2.2 − −0.7)	0.861
Boys (*p* < 0.001 **)	−1.46 ± 1.277	−1.5 (−2.2 − −0.6)	

SD, standard deviation; IQR, interquartile range; * Mann–Whitney U Test; ** Shapiro–Wilk Test.

**Table 4:** Chronological age, dental age, and differences between the two for girls and boys.

Age Group (in Years)	CA with SD (in Years)	DA with SD (in Years)	CA-DA with SD (in Years)	*p* *
**Girls**				
3–3.9 (*p* = 0.253 **)	3.40 ± 0.36	3.86 ± 0.40	−0.46 ± 0.75	<0.001
4–4.9 (*p* = 0.100 **)	4.52 ± 0.35	6.00 ± 1.03	−1.47 ± 0.87	
5–5.9 (*p* = 0.220 **)	5.57 ± 0.23	7.05 ± 0.38	−1.47 ± 0.36	
6–6.9 (*p* = 0.198 **)	6.54 ± 0.28	8.20 ± 0.66	−1.65 ± 0.69	
7–7.9 (*p* < 0.001 **)	7.55 ± 0.28	8.75 ± 0.86	−1.08 ± 1.32	
8–8.9 (*p* = 0.020 **)	8.42 ± 0.30	9.52 ± 1.13	−1.1 ± 1.1	
9–9.9 (*p* = 0.186 **)	9.42 ± 0.30	10.92 ± 1.31	−1.5 ± 1.21	
10–10.9 (*p* = 0.003 **)	10.41 ± 0.28	11.97 ± 1.20	−1.55 ± 1.11	
11–11.9 (*p* = 0.001 **)	11.45 ± 0.29	13.19 ± 1.37	−1.73 ± 1.36	
12–12.9 (*p* = 0.016 **)	12.40 ± 0.28	13.73 ± 1.67	−1.32 ± 1.62	
13–13.9 (*p* < 0.001 **)	13.48 ± 0.33	15.03 ± 1.60	−1.55 ± 1.54	
**Boys**				
3–3.9 (*p* = - **)	3.25 ± 0.35	3.40 ± 0.56	−0.15 ± 0.21	0.152
4–4.9 (*p* = 0.110 **)	4.37 ± 0.21	5.24 ± 1.55	−0.87 ± 1.41	
5–5.9 (*p* = 0.058 **)	5.51 ± 0.32	7.03 ± 0.56	−1.51 ± 0.45	
6–6.9 (*p* < 0.001 **)	6.56 ± 0.30	8.23 ± 0.92	−1.66 ± 0.84	
7–7.9 (*p* < 0.001 **)	7.56 ± 0.28	9.10 ± 1.22	−1.53 ± 1.17	
8–8.9 (*p* = 0.017 **)	8.49 ± 0.30	9.96 ± 1.26	−1.46 ± 1.25	
9–9.9 (*p* = 0.608 **)	9.36 ± 0.30	10.69 ± 1.14	−1.32 ± 1.14	
10–10.9 (*p* = 0.003 **)	10.37 ± 0.29	12.40 ± 1.25	−2.02 ± 1.24	
11–11.9 (*p* = 0.182 **)	11.40 ± 0.29	12.59 ± 1.52	−1.19 ± 1.53	
12–12.9 (*p* = 0.063 **)	12.44 ± 0.31	13.97 ± 1.61	−1.52 ± 1.57	
13–13.9 (*p* = 0.002 **)	13.40 ± 0.29	14.72 ± 1.51	−1.32 ± 1.43	

CA, chronological age; DA, dental age; SD, standard deviation; * Kruskal–Wallis H Test, ** Shapiro–Wilk Test.

## Data Availability

The data presented in this study are available on request from the corresponding authors. The data are not publicly available due to privacy reasons.
